# History and Perspective of LAMP-2 Deficiency (Danon Disease)

**DOI:** 10.3390/biom14101272

**Published:** 2024-10-09

**Authors:** Kazuma Sugie, Ichizo Nishino

**Affiliations:** 1 Department of Neurology, School of Medicine, Nara Medical University, Nara 634-8521, Japan; ksugie@naramed-u.ac.jp; 2 Department of Neuromuscular Research, National Institute of Neuroscience, National Center of Neurology and Psychiatry (NCNP), Tokyo 187-8551, Japan

**Keywords:** Danon disease, LAMP-2, autophagy, cardiomyopathy, myopathy, autophagic vacuole, lysosome

## Abstract

Danon disease, an X-linked dominant vacuolar cardiomyopathy and skeletal myopathy, is caused by a primary deficiency of lysosome-associated membrane protein-2 (LAMP-2). This disease is one of the autophagy-related muscle diseases. Male patients present with the triad of cardiomyopathy, myopathy, and intellectual disability, while female patients present with cardiomyopathy. The disease’s leading cause of death is heart failure, and its prognostic factor is cardiomyopathy. Pathologically, the disease is characterized by the appearance of unique autophagic vacuoles with sarcolemmal features (AVSFs). Twenty-six families have been found to have this disease in Japan. It has been over 40 years since the first report of this disease by Danon et al. and over 20 years since the identification of the causative gene, *LAMP2*, by Nishino et al. Although the pathogenetic mechanism of Danon disease remains unestablished, the first clinical trials using AAV vectors have finally begun in recent years. The development of novel therapies is expected in the future.

## 1. Concept and History of Danon Disease

Danon disease (MIM# 300257) is an extremely rare myopathy, first described as a “lysosomal glycogen storage disease with normal acid maltase” by Danon al. in 1981 [1]. In 2000, Nishino et al. revealed a primary deficiency of LAMP-2 caused by a mutation in *lysosome-associated membrane protein type 2* (*LAMP2*) [2]. Subsequently, in 2002, the authors reported for the first time that Danon disease showed X-linked dominant inheritance, with hypertrophic cardiomyopathy, myopathy, and intellectual disability as the triad of clinical symptoms in males and cardiomyopathy as the main symptom in females [3]. It is an extremely rare disease, with about 100 families found so far in the world [4] and 20 families found in a nationwide survey in Japan [5]. With the addition of six newly identified families reported after the survey, we have found 26 families in Japan. The cardiomyopathy of Danon disease is severe and is a prognostic factor for life. It has recently attracted attention as a differential diagnosis for hypertrophic cardiomyopathy of unknown cause [6,7,8], particularly if symptoms other than cardiomyopathy are mild.

Although it has been over 40 years since the first report of Danon disease, the path to understanding its underlying mechanisms remains rocky. Indeed, the clinical outcome for patients who do not receive heart transplantations is extremely bleak. However, findings from LAMP-2-deficient animal models and patient-derived induced pluripotent stem cells have shed light on this pathway, and promising results from gene therapy point us in the direction of future treatments. We are confident that we will be able to improve its prognosis and overcome early death in the near future [9,10].

## 2. Clinical Symptoms

Danon disease is an X-linked dominant inheritance, and about half of the mothers of boys with the disease are symptomatic. The remainder are considered *de novo* mutations. Male patients present with the triad of cardiomyopathy, myopathy, and intellectual disability, while female patients present primarily with cardiomyopathy [3,4,5,6,11]. In males, onset occurs in teenage years and death occurs around age 30, while onset occurs later in females than in males, typically after age 30, and death occurs around age 40 [12]. Recently, early-onset female patients have been observed [6]. Both sexes inevitably present with myocardial damage and hypertrophic cardiomyopathy [8,13]. A high rate of cardiac conduction defects, such as Wolff–Parkinson–White syndrome, complicate the disease. Myopathy is characterized by muscle weakness and atrophy with a predominance of proximal muscles. Intellectual disability is present in about 60% of patients but is mild. In some patients, small vessel lesions in coronary arteries and cerebral arteries have been observed, with vascular stenosis caused by proliferation of vascular smooth muscle cells due to autophagy abnormalities [14].

## 3. Muscle Pathology

**Figure 1:**
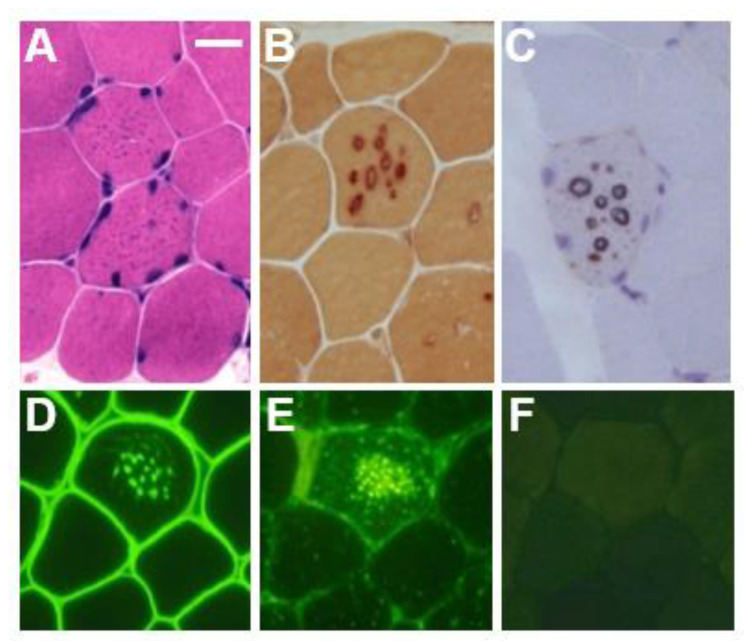
Muscle pathology in a male patient with Danon disease. Basophilic tiny autophagic vacuoles (arrows) are scattered in muscle fibers stained with hematoxylin and eosin (**A**). The small vacuoles have acid phosphatase activity (**B**) and acetylcholine esterase activity (**C**). Immunohistochemical staining shows the expression of sarcolemmal proteins such as dystrophin (**D**) in the autophagic vacuolar membrane (AVSF). LIMP-1, a marker of lysosomes, is strongly expressed (**E**) and LAMP-2 is absent (**F**). A–F: Bar = 30 μm; D–F: serial sections. With permission from our previous report [5].

**Figure 2:**
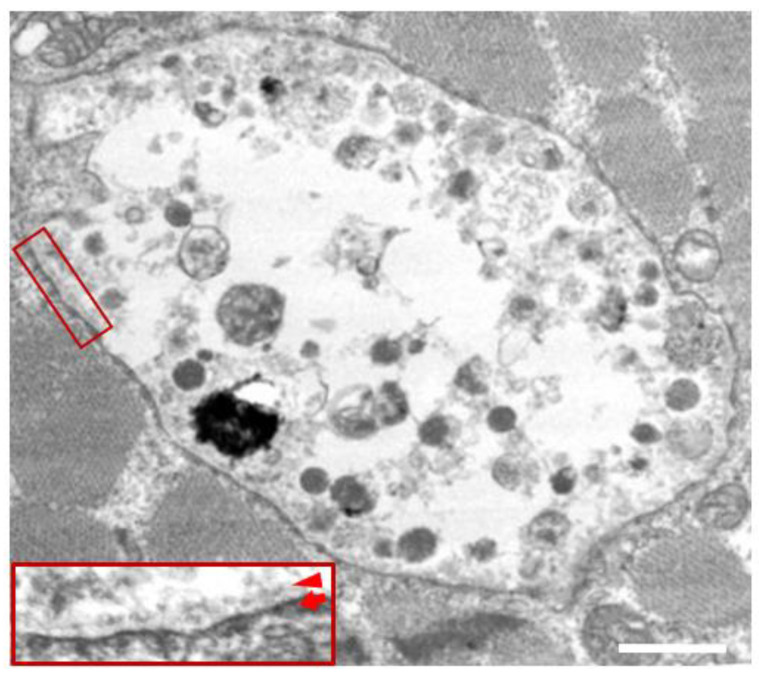
Electron microscopy in the skeletal muscle of a male patient with Danon disease. The vacuoles have autophagic nature as indicated by the presence of electron-dense granular materials, myeloid bodies, and variable cytoplasmic debris. In addition, basal lamina (red arrowhead) is likely to be observed along the inner surface of an autophagic vacuole (red arrow), suggesting further evidence that the vacuolar membrane has sarcolemmal features. Bar = 1 nm. Published with permission from our previous report [5].

In the biopsied muscles of individuals with Danon disease, muscle fibers with small vacuoles are scattered throughout the muscle fascicles. The membranes of these small vacuoles show acetylcholine esterase (AChE) activity and the expression of sarcolemmal proteins such as dystrophin and sarcoglycan, which are normally found only at the neuromuscular junction (Figure 1). We call this autophagic vacuole with sarcolemmal proteins (AVSF) [15,16]. Our observations show the formation of AVSF to be a phenomenon in which cells create an extracellular environment inside themselves, but its mechanism and involvement in the disease’s pathogenesis remain unclear. Electron microscopic observations reveal a basement membrane along the inside of the vacuolar wall, characterized by autophagic vacuoles containing irregularly shaped abnormal structures and glycogen granules (Figure 2).

Immunohistochemical and western blot analyses reveal the complete absence of LAMP-2 protein in skeletal muscle and cardiac muscle regardless of a specific *LAMP2* gene mutation in male patients [3,15] (Figure 1). Other lysosomal membrane proteins, such as lysosomal integral membrane protein-I (LIMP-1), are associated with the autophagic vacuoles in Danon disease [3,15]. Western blot analysis of LAMP-2 staining for females differs from that of male patients. Normal LAMP-2 expression has been observed in some female patients [16,17], and two female patients showed decreased LAMP-2 staining (40%), most likely due to ‘LAMP-2 haploinsufficiency’ [5,6,11], suggesting that this test may be inconclusive in females. On the other hand, the deletion of LAMP-2 protein was observed in the muscle of a few girls with early onset myopathy and cardiomyopathy [18], which may reflect an extremely skewed X-chromosome inactivation pattern (XCI) that favors the mutant allele. In fact, successful XCI pattern–disease severity correlations have been observed in females [19,20,21]. More recently, the irregular distribution of LAMP-2 in cardiac muscle fibers has been raised as a major determinant of the development of cardiomyopathy in females [22,23].

## 4. Biochemical and Genetic Characteristics of LAMP-2 and Autophagy

LAMP-2 is a component of the lysosomal membrane and, together with its complement LAMP-1, may protect the lysosomal membrane and cytoplasm from proteolytic enzymes in the lysosomal lumen. In contrast to the constant expression of LAMP-1, the expression of LAMP-2 increases under various conditions. *LAMP2* is composed of nine exons, with exons 1–8 and part of exon 9 constituting the lumenal domain and the remainder of exon 9 constituting both the transmembrane and cytoplasmic domains. Selective splicing of exon 9 results in 9A/9B/9C, with three isoforms, LAMP-2A/2B/2C, respectively. LAMP-2A is universally expressed in tissues, while LAMP-2B is known to be expressed mainly in the cardiac muscle, skeletal muscle, and brain and may play an important role in the pathogenesis of this disease. The role of LAMP-2C is still unclear. In Danon disease, autophagy, which occurs constantly in these regions, is thought to cease at the final stage of fusion between autophagosomes and lysosomes [24].

More recently, in addition to autophagy dysfunction, the involvement of mitophagy defects, oxidative stress, and energy deficiency have been reported to play important pathophysiological roles in the development of Danon disease [25,26,27,28,29]. Dysfunction of mitophagy may be an early pathological hallmark of Danon disease with important upstream effects such as impaired mitochondrial metabolism. These results suggest a link between lysosomal and autophagic dysfunction due to LAMP-2 deficiency and mitochondrial turnover defects [27]. Indeed, the downregulation of main mitophagy target genes at the mRNA level was evident. Moreover, mitochondria were partially co-localized with p62+ vacuoles, suggesting a hallmark of Danon disease—the impaired lysosomal clearance of dysfunctional mitochondria [29,30]. As a common final pathway for non-selective autophagy as well as mitophagy, dysfunctional lysosomes may inhibit mitochondrial degradation after fusion with autophagosomes [31].

**Figure 3:**
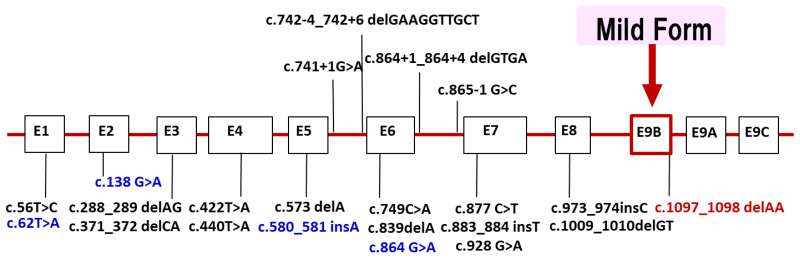
*LAMP2* mutations in Japanese patients with Danon disease. We identified 22 different *LAMP2* mutations in 26 families with Danon disease. Four mutations (blue) were identified after the nationwide survey in 2018. The distribution of mutations widely ranged from exon 1 to 9. Four families with mutations in exon 9B (red) which encodes LAMP-2B showed markedly mild or no cardiomyopathy. Exons are to scale and introns are not to scale.

*LAMP2* is located in Xq24. Most of the reported mutations are stop codons or out-of-frame, which are thought to cleave the protein and cause loss of the transmembrane and cytoplasmic domains. About 100 families [4] have been found so far in the world, and in Japan’s nationwide survey conducted by the authors in 2018, 20 families were found [5]. We have added patients newly found and reported since the 2018 national survey in Japan. Currently, we have identified 22 different *LAMP2* mutations in 26 families (Figure 3). Half of the probands showed de novo mutations. The distribution of mutations widely ranged from exon 1 to 9. We found four families with mutations in exon 9B (c.1097_1098 delAA) that encodes LAMP-2B, which is considered genetically important. Recently, we reported that male patients over 50 years of age with mutations in exon 9B showed very mild clinical symptoms, including cardiomyopathy [32]. Our findings suggest the presence of factors in exon 9B that may prevent severe clinical symptoms, but they have not yet been established.

## 5. Definitive Diagnosis

There are no internationally established diagnostic criteria for Danon disease. However, diagnostic criteria for Danon disease were developed by the “Autophagocytic Vacuolar Myopathy Study Group” and the “Study Group on Rare and Intractable Muscle Diseases” of the Japanese Ministry of Health, Labour and Welfare, taking into consideration the necessary and sufficient conditions for diagnosis (Table 1). The diagnostic criteria were approved by the Japan Society of Neurology. The diagnosis of Danon disease is confirmed by clinical features and muscle pathology, as well as LAMP-2 deficiency in biopsied muscle and/or western blot analysis, and *LAMP2* gene analysis. In female patients, LAMP-2 protein varies from deficient to normal, but LAMP-2 haploinsufficiency is also considered as a contributing factor [6,11,15]. As Danon disease is extremely rare, it is practically difficult to suspect Danon disease and perform genetic analysis based on clinical symptoms alone. For diagnosis, it is important to perform a muscle biopsy and find AVSF in the muscle fibers. In fact, the three main criteria for diagnosis are (A) clinical features; (B) muscle pathology; and (C) evaluation of LAMP-2. A “definite diagnosis” is defined as a patient who meets at least one of criteria A or B and also meets criterion C. Clinically, differential diagnosis includes other myopathies and muscular dystrophies and cardiomyopathies of other definite causes. Pathologically, it is important to differentiate the diseases from other muscle diseases with autophagic vacuoles.

As radical treatment has yet to be established for Danon disease, early diagnosis is essential for the planning of appropriate treatments to prevent sudden cardiac death prior to heart transplantation [12,33,34,35,36]. In fact, the frequency of Danon disease is 4–6% in children with hypertrophic cardiomyopathy [33], suggesting that it is one of the major causes of hypertrophic cardiomyopathy. Given that most patients die of heart failure or arrhythmia, palpitations or chest pain are prominent clinical features and the most important prognostic factors. Heart transplantation significantly improves survival, but only 17.6% of patients with Danon disease undergo this procedure [12]. Therefore, disease awareness and early diagnosis of Danon disease are critical.

## 6. Treatment and Prognosis

In Danon disease, cardiomyopathy is the determinant of life prognosis, and the cause of death is sudden death due to heart failure or cardiac conduction defects [7,8,15]. Currently, the only radical therapy is heart transplantation, and catheter ablation or implantable cardioverter defibrillators should be considered at the early stages of the disease [37]. Since transplantation is desired within two years of the onset of heart failure, early diagnosis is required. Furthermore, since there is a possibility that Danon disease patients are hidden among patients with hypertrophic cardiomyopathy of unknown cause, genetic analysis should be considered early on if this disease is suspected. In addition, since female patients with Danon disease have only cardiomyopathy and no or minimal skeletal muscle symptoms, an undiagnosed family history of the disease may be considered. We suggest further investigations of relatives who are at risk of developing the disease and believe that careful follow-up observation is particularly important for asymptomatic adult women. As there is a risk of sudden death in female patients, it is necessary to consider the application of catheter ablation or defibrillators, as well as early drug therapy. The degree of intellectual disability is mild and not life-threatening, but it is necessary to consider providing support for patients’ learning and mental health, as well as education. This can include referring patients to local intellectual disability support centers and connecting them with relevant mental health professionals.

Since this disease is extremely rare, the accuracy of the current diagnostic criteria will need to be verified through an accumulation of patients and collaboration with other medical departments. Furthermore, future issues in the policy of intractable diseases include nationwide epidemiological surveys, clarification of natural history based on the results of these surveys, identification of new patients, and the establishment of a registry.

## 7. Research for Novel Therapeutic Strategies of Danon Disease

Vigorous efforts are now being made on various fronts to develop new treatment methods. An initial step was to use animal models. *LAMP2* knockout (KO) mice generated by a German group demonstrated that LAMP-2 deficiency causes Danon disease [24]. Approximately 50% of *LAMP2* KO mice died between 20 and 40 days of age, regardless of sex or genetic background. *LAMP2* KO mice showed autophagic vacuoles in various organs, including the heart, skeletal muscle, liver, pancreas, spleen, and kidney; the surviving mice were small and showed cardiac hypertrophy. KO mice that died early often showed small intestinal stenosis and pancreatic lesions, which may be the result of the KO mice showing multiorgan involvement. This suggests that Danon disease patients may develop multiorgan involvement in addition to the triad of signs. On the other hand, *LAMP1* deficient mice show normal lysosomal morphology and function and do not develop any symptoms [38]. This suggests that LAMP-1 and LAMP-2, which are homologous to each other, may play different roles, as LAMP-2 serves a compensatory hyperfunction in *LAMP1* KO mice whereas LAMP-1 is not hyperfunctional in *LAMP2* KO mice [24].

Subsequently, rat models of Danon disease were developed in 2017 and zebrafish models were developed in 2019 [39,40]. These models showed the phenotypes of Danon disease, including cardiomyopathy and myopathy and the presence of autophagic vacuoles. In addition, mTOR inhibition was shown to normalize features of the *LAMP2* KO, including ejection fraction, β-adrenergic response, and actomyosin activation kinetics, demonstrating the potential of new intervention strategies related to mTOR [40]. Breakthroughs in patient-derived pluripotent stem cell technology have opened new avenues for the elucidation of genetic diseases, including Danon disease, and it has been used as a powerful tool [10]. Using a small amount of blood collected from patients, human induced pluripotent stem cell-derived cardiomyocytes (hiPSC-CM) were cultured to provide important insights into development. This technique has been widely utilized by research groups around the world and has contributed significantly to research advances [41]. In vitro and in vivo studies since 2018 have revealed the role of mitochondrial dysfunction and fragmentation in pathological mechanisms and potential targets for therapeutic intervention [14,28]. Notably, in patient-derived hiPSC-CMs, correction of the *LAMP2* mutation with single-stranded DNA using CRISPR-Cas9 resulted in increases in ATP production, oxygen consumption rate, and maximal respiratory rate comparable to those in normal hiPSC-CMs. These breakthrough results suggest that this correction may improve the partial phenotype of Danon disease in vitro [28].

On the other hand, recent years have also seen advances in the pharmacological aspects of Danon disease treatment strategies. Some studies show the efficacy of conventional drug therapies used to control heart failure and ventricular remodeling, mainly inhibitors of the renin–angiotensin–aldosterone system (RAAS), in particular Ramipril [42]. In fact, there have been several reports on the effects of RAAS on autophagy and its role in cardiac hypertrophy across various models [43]. However, the causal relationship between oxidative stress and cardiac phenotype and whether antioxidants play any role remain unclear.

In an in vivo study using *LAMP2* KO mice in 2020, Manso et al. reported an innovative method to correct LAMP-2B deficiency using recombinant adeno-associated virus (AAV9) as a vector [44]. They observed that human LAMP-2B was constructed on AAV9 and that LAMP-2B protein was persistently expressed; intravenous injection of AAV9-LAMP-2B into *LAMP2* KO mice resulted in dose-dependent recovery of LAMP-2 protein in the heart, liver and skeletal muscle. Survival of the gene-treated aged mice clearly improved [44]. Based on this study, the first clinical trials of promising gene therapy on human patients with Danon disease started in the United States in 2019 (NCT03882437) [45,46]. The trials were part of a nonrandomized, open-label phase 1 study to evaluate the safety and toxicity of gene therapy with AAV9-LAMP-2B (RP-A501). Secondary outcomes at the end of three years included assessments of cardiac and skeletal muscle gene transfer induced by therapy, histological evaluation of cardiomyocytes, and the clinical assessment of cardiomyopathy. As of 2024, phase 2 trials are underway (NCT06092034) [46].

In addition, recent studies have shown that the efficacy of the RAAS system with ramipril is equivalent to that of gene therapy, and the protective effect of drug combination therapy is expected to be even greater than that of ramipril alone, suggesting a need to extend the drug therapy that is currently applied to Danon disease patients with heart failure [47]. Further elucidation of the functions and molecular mechanisms of autophagy and lysosomes in Danon disease is expected to elucidate the disease’s pathomechanisms and lead to the development of therapeutic methods.

## 8. Conclusions

Over 40 years since the first report of Danon disease and over 20 years since the identification of its causative gene have passed, but heart transplantation remains the only curative treatment, and the path to elucidating the underlying pathophysiology is steep. However, dramatic advances have been made in clinical, genetic, and basic research over the past 25 years, and the pathophysiology caused by autophagy abnormalities is becoming clearer [48,49]. The postulated pathomechanism underlining Danon disease may be impaired mitochondrial metabolism due to dysfunction of mitophagy, as well as lysosomal and autophagy dysfunction due to LAMP-2 deficiency. After fusion with autophagosomes, dysfunctional lysosomes may inhibit mitochondrial degradation through non-selective autophagy and mitophagy. The development of model animals such as mice, rats, and zebrafish, as well as findings from patient-derived iPS cells, provide a ray of hope on Danon disease research. As these studies demonstrate, correction of the *LAMP2* mutation resulted in increases in ATP production, oxygen consumption rate, and maximum respiratory rate, all of which are comparable to normal in vitro, indicating that this correction may improve the partial phenotype of Danon disease and reveal potential targets for therapeutic intervention. Furthermore, the first clinical trials of promising gene therapy in humans finally began in 2019, with phase 2 trials currently underway as of 2024. We hope that the prognosis and survival rate will improve in the near future.

## Figures and Tables

**Table 1:** Diagnostic criteria for Danon disease. (Japanese Ministry of Health, Labour and Welfare, “Autophagic Vacuolar Myopathy Study Group” (2012)).

**A. Clinical features** ((a), (b) required for males, (a) required for females, and (c)–(g) are reference findings) (a) Hypertrophic or dilated cardiomyopathy (b) Progressive muscle weakness/atrophy or hyperCKemia **(Reference findings below)** (c) X-linked dominant inheritance or solitary (d) Age of onset is usually in the teens for males and in the 30s for females (e) Often accompanied by intellectual retardation (f) Serum CK level normal to mildly elevated (<1000 IU/L) (g) Myogenic changes (early recruitment and/or low amplitude of MUP) are observed on electromyography **B. Muscle pathology** ((a) and (b) are required; (c) and (d) are reference findings) (a) Myofibers with autophagic vacuoles (b) Increased acetylcholine esterase activity on the vacuolar membrane (histochemical staining in skeletal muscle) **(Reference findings below)** (c) Expression of sarcolemmal proteins (dystrophin, sarcoglycan, laminin-α2, caveolin-3, etc.) on the vacuolar membrane (immunohistochemical staining in the skeletal muscle) (d) Presence of a basement membrane around autophagic vacuoles (electron microscopy) **C. Evaluation of LAMP-2** ((a) or (b) is required) (a) LAMP-2 deficiency (immunohistochemistry or Western blot analysis) However, in female patients, decreased LAMP-2 proteins (b) *LAMP2* mutation **Diseases to be excluded** Clinical Differentiation Other muscle diseases such as myopathies and muscular dystrophies Cardiomyopathy with other confirmed causes Pathological Differentiation Other myopathies with autophagic vacuoles: Pompe disease, X-linked myopathy with excessive autophagy, GNE myopathy, inclusion body myositis, etc. **Diagnostic Category** Definite diagnosis: At least one of A or B is satisfied and C is satisfied. Possible diagnosis: A and B are satisfied; there is a definite diagnosis in the family and C is satisfied

## Abbreviations

AVSF	autophagic vacuoles with sarcolemmal features
CK	creatine kinase
LAMP-2	lysosome-associated membrane protein-2
LIMP-1	lysosomal integral membrane protein-1
